# The efficacy and safety of gemcitabine-based induction chemotherapy for locally advanced nasopharyngeal carcinoma treated with concurrent chemoradiation: A meta-analysis

**DOI:** 10.1097/MD.0000000000025398

**Published:** 2021-04-09

**Authors:** Qian Fei, Han-Bo Chen, Chun-Mei Zhang, Jia-Jun Xu, Xia He, Song-Wang Chen

**Affiliations:** a Nanjing First Hospital, Nanjing Medical University, Nanjing; b The Affiliated Cancer Hospital of Nanjing Medical University and Jiangsu Cancer Hospital and Jiangsu Institute of Cancer Research, 42 Bai Zi Ting Road, Nanjing, Jiangsu, China.

**Keywords:** gemcitabine, induction chemotherapy, meta-analysis, nasopharyngeal carcinoma

## Abstract

**Objectives:**

To assess the efficacy and toxicity of gemcitabine-based induction chemotherapy followed by concurrent chemoradiotherapy (CCRT) in locally advanced nasopharyngeal carcinoma (LA-NPC).

**Methods:**

Both observational studies (OBS) and randomized controlled trials (RCT) were included in the meta-analysis. Systematic online searches were conducted in Web of Sciences, PubMed, Embase, meeting proceedings and ClinicalTrials.gov from the inception to May 25, 2020. The primary endpoint of interest was overall survival.

**Results:**

five OBSs and 2 RCTs including 1680 patients were incorporated in the analysis. The evidence from the RCTs showed that adding gemcitabine-based induction chemotherapy to CCRT significantly improved progression free survival (hazard ratio (HR): 0.60, 95% confidence interval (CI): 0.40–0.88; *P* = .010; chi square *P* = .25; *I*^2^ = 24%) and overall survival (HR: 0.47; 95% CI: 0.28–0.80; P = 0.005; chi square *P* = .49, *I*^2^ = 0%) and was related to a higher risk of hematological toxicities. Furthermore, based on the data of OBSs, overall survival (HR: 0.52; 95% CI: 0.31–0.88; *P* = .02; chi square *P* = .37, *I*^2^ = 6%) was significantly improved in patients treated with gemcitabine-based induction chemotherapy compared to those treated with taxane-based induction chemotherapy. However, the progression free survival (HR: 0.67; 95% CI: 0.45–1.01; *P* = .06; chi square *P* = .74; *I*^2^ = 0%) showed no significant difference.

**Conclusions:**

For LA-NPC patients, adding gemcitabine-based induction chemotherapy to CCRT significantly improved overall survival and progression free survival with a higher risk of hematological toxicities when compared to CCRT alone. Also, gemcitabine-based regimen could be used as an alternative induction chemotherapy regimen to taxane-based regimen in the treatment of LA-NPC.

## 1 Introduction

Nasopharyngeal carcinoma (NPC) is a relatively rare tumor with uneven geographical distribution and high morbidity in local areas, especially in Southeast Asia.^[1]^ According to the International Agency for Research on Cancer, approximately 129,000 cases of NPC were newly diagnosed in 2018, accounting for 0.7% of all cancers.^[2]^ Intensity-modulated radiotherapy significantly improved five-year overall survival (OS) of locally advanced nasopharyngeal carcinoma (LA-NPC) patients.^[3]^ Concurrent chemoradiotherapy (CCRT) is the standard treatment for LA-NPC.^[4,5]^ Given the poor prognosis of patients with LA-NPC, chemotherapy plays a crucial role in the treatment of high-risk NPC patients.^[6–8]^ Recently, several multicenter clinical trials have shown that induction chemotherapy (IC) followed CCRT can significantly improve the prognosis of LA-NPC patients.^[9–16]^ In addition, the National Comprehensive Cancer Network (Version 1. 2018) increased the category of evidence for IC plus CCRT from 3 to 2A in the treatment of LA-NPC.^[17]^ Therefore, we can consider that IC plus CCRT might be a promising therapeutic strategy for LA-NPC patients. However, up to now the optimal IC regimen for LA-NPC has not been established.

Several IC regimens, including TP (docetaxel, cisplatin), FP (cisplatin, fluorouracil), and TFP (docetaxel, cisplatin, fluorouracil), are known to improve the survival of patients with NPC.^[18,19]^ A multicenter randomized phase III trial showed that the efficacy of gemcitabine combined with cisplatin (GP) as IC for recurrent or metastatic NPC was similar to that of TFP, and the incidence of 3 or 4 grade adverse events (AEs) was significantly reduced in GP group.^[19]^ Moreover, gemcitabine has showed high therapeutic efficacy in various tumor types.^[20–22]^ Recently, a multicenter, randomized, controlled, phase 3 trial for LA-NPC demonstrated that the addition of IC to CCRT significantly improved OS and recurrence-free survival (RFS), when compared with CCRT alone.^[23]^ Currently, the National Comprehensive Cancer Network (Version 2. 2020) mentions cisplatin and gemcitabine as category 1 recommendation of IC regimen for NPC patients.^[24]^ But now, whether GP is an ideal IC regimen remains controversial and no pooled analysis has been conducted to assess the clinical effects of GP-based IC added to CCRT in LA-NPC.

In this meta-analysis, the therapeutic effect was evaluated by incorporating randomized controlled trials (RCT) and observational studies (OBS). RCT studies have eliminated confounders in the study environment to a certain extent due to the use of randomization, blinding, and control principles, thus becoming the “gold standard” for the evaluation of causal effects in clinical studies.^[25]^ It is known that OBS is a kind of real-world study, and the research environment is closer to the actual clinical environment, thus proving its high external validity.^[25]^

## 2 Material and methods

This meta-analysis was performed based on the Preferred Reporting Items for Systematic Reviews and Meta-Analyses (PRISMA).^[26]^ No ethical approval was needed and all included studies have been published.

### 2.1 Search strategy and selection criteria

Systematic online searches were conducted in Web of Sciences, PubMed, Embase, ClinicalTrials.gov, and meeting proceedings from the inception to May 25, 2020. The following Medical Subject Headings (MeSH) term were applied:(“Nasopharyngeal Carcinoma”) AND (“Induction Chemotherapy”). Additionally, we searched the reference lists of included studies, review papers, and meeting proceedings to identify other relevant studies as supplement.

Inclusion criteria:

(1) participants [P]: Patients diagnosed with NPC (stages III–IV, American Joint Committee on Cancer staging system);
(2) intervention [I]: GP followed by CCRT;
(3) comparison [C]: taxane-based IC plus CCRT or CCRT alone;
(4) study design [S]: RCT or OBS;
(5) outcomes [O]: safety and efficacy.

Exclusion criteria:

(1) not included interventions of interest;
(2) review:
(3) unable to retrieve full articles;
(4) no comparison group;
(5) insufficient data;
(6) likely duplicate reports.

### 2.2 Risk of bias assessment

The Cochrane collaboration risk of bias assessment tool assessed the risk of bias in RCTs in terms of random sequence generation, blindness, incomplete outcome data, allocation concealment and selective reporting. Based on the description of a previous study by Wang,^[27]^ the Newcastle-Ottawa Scale (NOS) was used to assess the quality of OBS. In the meta-analysis, funnel plots were not used to assess the likelihood of publication bias because of the insufficient number of trials.

### 2.3 Data extraction and checking

The data for each patient were independently extracted from literature by 2 partners, which included year of publication, the number of patients, first author's last name, basic information and intervention measures, OS, progression free survival (PFS), and adverse events (AEs). We checked the data in accordance with the standard procedures. Any differences were resolved by consensus.

### 2.4 Outcomes

The observational outcomes of this meta-analysis included OS, PFS. The primary endpoint was OS. For RCTs, OS was defined as the date from randomization to death. As secondary outcome, PFS was calculated from random grouping to disease recurrence or death. AEs, such as neutropenia, leukopenia, and thrombocytopenia, were included as important secondary outcomes.

### 2.5 Statistical analysis

RevMan software version 5.3 (Cochrane compact, Oxford, UK) was used for data analysis. All data were extracted directly from the study. Hazard ratio (HR), and 95% confidence interval (CI) were calculated as the result of effect quantity representation. Event time endpoints (OS and PFS) were summarized using HR, and HR<1 suggested that GP+CCRT treatment yielded a better survival rate than the control group. The incidence of treatment-related AEs was assessed on the basis of the relative odds (RR). Cochran *Q* test and *I*^2^ statistics were used to evaluate heterogeneity, in which heterogeneity was quantified rather than the proportion of total variation due to randomization.^[28]^ Heterogeneity was classified as low (*I*^2^ value between 25%–50%), medium (*I*^2^ value between 50%–75%), or high (*I*^2^ value between > 75%). For low evident heterogeneity, a random-effect model was applied for summary estimation. On the contrary, if the *I*^2^ value was more than 50% or the *P* value of the Cochrane *Q* test was less than 0.10, which implied obvious heterogeneity existed among the studies, a fixed-effects model was used.^[29,30]^

## 3 Results

### 3.1 Study search

**Figure 1:**
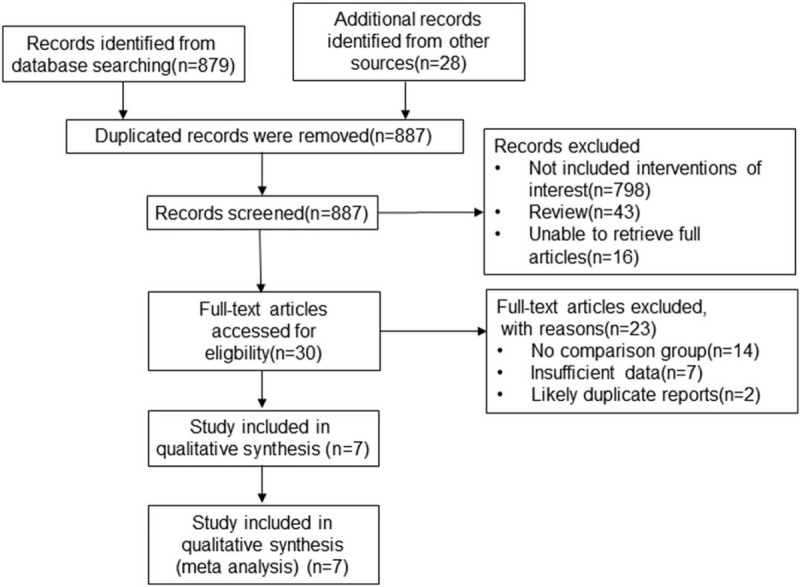
PRISMA flow chart of study identification and selection.

A total of 907 publications were screened by searching PubMed, Web of Sciences, Embase, ClinicalTrials.gov, and meeting proceedings from the research inception to May 25, 2020. After eliminating the duplications, 887 studies were left. By reading the full text, seven eligible studies were screened, including 1680 patients with low risk of bias in their methodological quality. Figure 1 shows the research screening flow diagram of meta-analysis.

### 3.2 Characteristics of included trials

Among the seven eligible studies, two were RCTs,^[23,31]^ and 5 were OBSs.^[19,32–35]^ After assessment, these seven studies were identified as high quality. Table 1 shows the main characteristics and related data included in these studies.

**Table 1:** Main features and data included in the meta-analysis.

Study	Trial phase	No. of patient	Median age	Median Follow-up	Stage	RT dose fractionation to gross disease	Agent for IC	Cycles of IC	Agent for CCRT	RTtechnique	Global score
Randomised trials											
Tan2016	II/III	IC+CCRT:86 CCRT:86	IC: 48.5 CCRT:51.6	IC: 40.8 months CCRT: 38.4 mo	III–IVB	IMRT:69.96 Gy/33f 2D RT:70 Gy/35f	gemcitabine (1000 mg/m^2^),paclitaxel (70 mg/m^2^,D1,8),carboplatin	3	cisplatin (40 mg/m^2^), 8 weeks	IMRT or 2D-RT	
Zhang2019	II	IC+CCRT:242 CCRT:238	IC: 46 CCRT:45	42.7 mo	III–IVB	NA	gemcitabine (1000 mg/m^2^, D1,8), cisplatin (80 mg/m^2^, D1)	3	cisplatin (100 mg/m^2^), 3 cycles	IMRT	
Observational studies											
Liu2018		TP:52 GP:52	49	60 mo	III–IVB	66–70 Gy/30–33f	GP: cisplatin ( 25mg/m^2^, D1–3), gemcitabine (1,000 mg/m^2^, D1,8); TP: cisplatin (25 mg/m^2^/day, D1–3 )and docetaxel (75 mg/m^2^/day, D1)	1–4	cisplatin (80–100 mg/m^2^), 1–2 cycles	IMRT	8
Zeng2018		TPF:58 GP:55	TPF:45.9 GP:48.2	51.4 mo	III–IV	66–74Gy/33–35f	TPF: docetaxel (60mg/m^2^, D1), cisplatin (75mg/m^2^, D1 or within 3 d), and 5-FU (600mg/m^2^, D1–5); GP: gemcitabine (1000mg/m^2^, D 1,8), cisplatin (75mg/m^2^, D 1 or within 3 d)	2–3	N	IMRT	6
Zheng2015		TP:444 GP:13	N	65 mo	III–IVB	NA	TP regimen: taxol (135 mg/m^2^, D1), cisplatin (80 mg/m^2^, D1–3); GP: gemcitabine (1000 mg/m^2^, D1,8),cisplatin (80 mg/m^2^, D1–3)	2	cisplatin (80–100 mg/m^2^,D1–3) or taxol (135 mg/m^2^,D1)+cisplatin (80 mg/m^2^,D1–3) or gemcitabine (1000 mg/m^2^,D1,8)+ cisplatin (80 mg/m^2^,D1–3) and fluorouracil (800 mg/m^2^,D1–5) + cisplatin (80 mg/m^2^,D1–3)	conventional radiotherapy or IMRT	6
Zhu2019		TPF:87 GP:71	TPF:45 GP:48	36 mo	III-IVA	70–74 Gy/33f	GP: gemcitabine (1000 mg/m^2^, D1,8), cisplatin (25 mg/m^2^, D1–3) TPF: docetaxel (l60 mg/m^2^, D1), cisplatin (20–25 mg/m^2^, D1–3), fluorouracil (600 mg/m^2^)	1–3	cisplatin (100 mg/m^2^, D1 or 25 mg/m^2^, D1–3)	IMRT	8
Zang2020		TP:142 GP:54	TP:51 GP:52	60.5 mo	III–IV	72.6 Gy/33f	GP: gemcitabine (1000 mg/m2, day 1 and day 8) plus cisplatin (75 mg/m2, day 1) every 3 wk for 2–3 cycles; TP: docetaxel (75 mg/m2, day 1) plus cisplatin (75 mg/m2, day 1)	2–3	cisplatin (100 mg/m^2^)	IMRT	7

2D-RT = 2D-radiotherapy, CCRT = concurrent chemoradiotherapy, GP = gemcitabine and cisplatin, IC = induction chemotherapy, IMRT = intensity-modulated radiotherapy treatment, NA = not available, RT = radiotherapy, TP = Docetaxel and cisplatin, TPF = docetaxel, cisplatin, and fluorouracil.

#### 3.2.1 RCT

**Figure 2:**
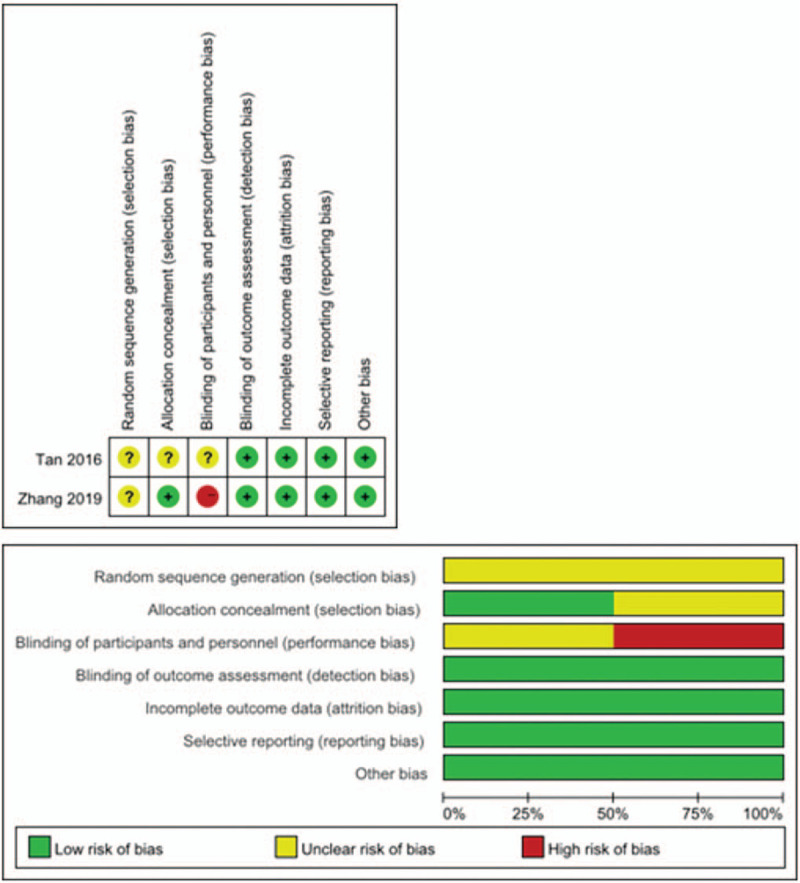
Risk of bias assessment for the randomized controlled trials.

A total of 652 patients with stage III–IVB NPC were enrolled in the two RCTs. The two studies investigated gemcitabine-based IC plus CCRT versus CCRT alone. The median age range was 45 to 51.6 years. All patients received cisplatin during radiotherapy (RT). The Cochrane collaboration risk of bias assessment tool was used to assess the risk of bias, and both trials were determined to be of high quality (Fig. 2).

#### 3.2.2 OBS

Five retrospective cohort studies included 1028 patients with stage III–IV NPC, which investigated GP plus CCRT vs taxane-based IC plus CCRT. The median age of the five studies was 45 to 51.5 years. Taxane-based IC regimens included TP and docetaxel, cisplatin, and fluorouracil. All patients received cisplatin-based chemotherapy during RT.

### 3.3 Efficacy on OS

#### 3.3.1 RCT

**Figure 3:**
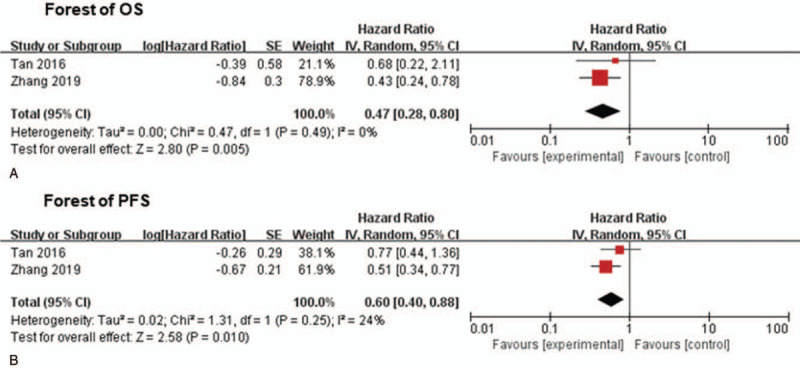
(A), Comparison of the OS of different type of radiotherapy: CCRT alone, and adding gemcitabine-based IC to CCRT (B), Comparison of the PFS of different type of radiotherapy: CCRT alone, and gemcitabine-based IC followed by CCRT. (Random effects forest plot). CCRT = concurrent chemoradiotherapy, IC = induction chemotherapy, OS = overall survival.

The results of analysis showed that compared to CCRT alone, adding gemcitabine-based IC to CCRT significantly improved OS (HR: 0.47; 95% CI: 0.28–0.80; *P* = .005; Fig. 3A). The results of *Q* test and *I*^2^ test on HRs showed no significant heterogeneity between the studies (chi square *P* = 0.49, *I*^2^ = 0%).

#### 3.3.2 OBS

**Figure 4:**
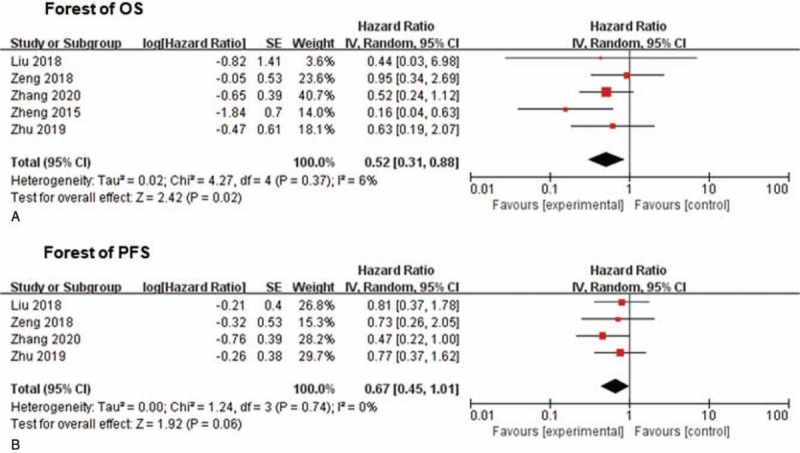
(A), Results (forest plot) of meta-analysis of OS of different type of radiotherapy: taxane-based IC plus CCRT, and GP followed by CCRT (B), Results (forest plot) of meta-analysis of PFS of different type of radiotherapy: taxane-based IC plus CCRT, and GP followed by CCRT. (Random effects forest plot). CCRT = concurrent chemoradiotherapy, GP = gemcitabine and cisplatin, IC = induction chemotherapy, OS = overall survival, PFS = progression free survival.

Comparing with patients who were treated with taxane-based IC plus CCRT, patients treated with GP followed by CCRT benefited with significantly longer OS (HR: 0.52; 95% CI: 0.31–0.88; *P* = .02; Fig. 4A). No heterogeneity was observed, confirming the validity of the pooled data (chi square *P* = .37, *I*^2^ = 6%).

### 3.4 Efficacy on PFS

#### 3.4.1 RCT

When compared to CCRT alone, gemcitabine-based IC followed by CCRT provided a significantly longer PFS for LA-NPC patients (HR 0. 60, 95% CI 0. 40–0.88; *P* = .010; Fig. 3B). Also, the results of *Q* and *I*^2^ tests in HRs showed low heterogeneity between the studies (chi square *P* = .25; *I*^2^ = 24%).

#### 3.4.2 OBS

Only four of the five OBSs reported PFS.^[19,32–34]^ There was a trend towards PFS benefit (HR: 0.67; 95% CI: 0.45–1.01; *P* = .06; Fig. 4B) in LA-NPC patients treated with gemcitabine-based IC plus CCRT. No heterogeneity was found among the included OBSs (chi square *P* = .74; *I*^2^ = 0%).

#### 3.4.3 AEs

**Figure 5:**
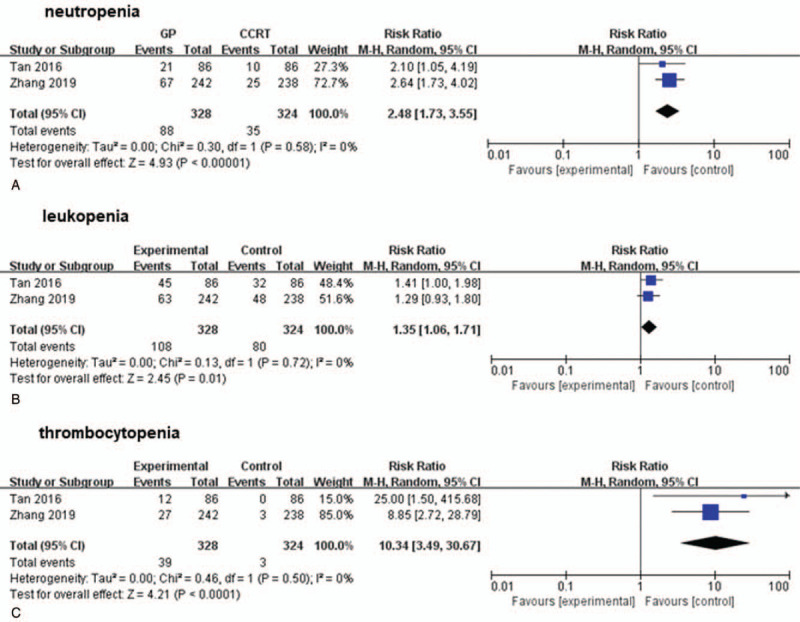
Compaison of the risk of hematological toxicities of different type of radiotherapy:GP followed by CCRT, and CCRT alone, (A), neutropenia (B), leukopenia (C), thrombocytopenia. CCRT = concurrent chemoradiotherapy, GP = gemcitabine and cisplatin.

Considering the reliability of data on AE in RCT and the inaccuracy of AE status in OBS, only RCT studies were analyzed. The results of analysis indicated that gemcitabine-based IC plus CCRT was significantly related to an increased risk of hematological toxicities, such as neutropenia (RR = 2.7, 95% CI:1.73–3.55, *P *< .0001; *I*^2^ = 0%, *P* = .58; Fig. 5A), leukopenia (RR = 1.35, 95% CI:1.06–1.71, *P* = .01; *I*^2^ = 0%, *P* = .72; Fig. 5B), and thrombocytopenia (RR = 10.34, 95% CI:3.49–30.67, *P* < .0001; *I*^2^ = 0%, *P* = .50; Fig. 5C). Besides, no significant heterogeneity was observed in Figure 5.

## 4 Discussion

This meta-analysis showed that adding gemcitabine-based IC to CCRT significantly improved PFS and OS in LA-NPC when compared with CCRT alone. Meanwhile, based on the results of the RCT studies, gemcitabine-based IC was related to an increased risk of hematological toxicities. To the best of our knowledge, this meta-analysis is the first to directly compare gemcitabine-based IC plus CCRT with CCRT alone or taxane-based IC plus CCRT.

Whether IC is needed and which type of IC regimen should be used have always be controversial in the treatment of LA-NPC patients. In 2015, a meta-analysis of four RCT studies conducted by Song showed that compared to CCRT alone, IC plus CCRT could significantly reduce the risk of distant metastasis (40%) and progression (34%) in LA-NPC, but of no significant OS benefit (HR = 0.52, 95% CI 0.21–1.29).^[36]^ Whereas, our study demonstrated that the addition of gemcitabine-based IC to CCRT significantly improved OS (HR: 0.47; 95% CI: 0.28–0.80; P = 0.005; Fig. 3A) when compared to CCRT alone. The reason for the different result of OS analysis might be that taxane-based IC regimen was used in Song's study.

On the basis of five OBS studies, our study suggested that OS was significantly improved in patients treated with gemcitabine-based IC compared to those treated with taxane-based IC (HR: 0.52; 95% CI: 0.31–0.88; *P* = .02; Fig. 4A). Similarly, Zheng found that for LA-NPC patients, gemcitabine-based IC was an independent prognostic factor for OS, whereas TP was only a significant predictive factor for metastasis-free survival.^[35]^ In 2018, Li et al has conducted a meta-analysis of 13 RCTs and the results suggested that compared to FP regimen, GP regimen might be considered as a better choice for advanced NPC patients without differences in toxicity.^[37]^ This study investigated GP as a chemotherapy regimen for distant metastasis or recurrence NPC patients with no opportunity of salvage treatment or surgery. Our research, however, found that GP regimen could be used as an alternative IC regimen to taxane-based regimen in the treatment of LA-NPC. Although the two meta-analyses were performed based on different subgroups of NPC patients, the results indicated that gemcitabine-based chemotherapy regimen might be a good choice both for recurrence or distant metastasis NPC and for LA-NPC.

This analysis showed the high number of 3 to 4 level treatment-related toxicity events that occurred in the GP group, especially thrombocytopenia. However, in different patients receiving chemotherapy, a considerable heterogeneity was observed in adverse drug reaction. After prophylactic treatment, the incidence of serious bleeding complications decreased. The proper prophylactic use of colony-stimulating factors reduces the relative risk of infection and infection-related mortality.^[38–40]^ Although AEs increased, gemcitabine-based IC regimen is considered to be less toxic than TFP, making the former an ideal option for IC.^[41]^

Although this meta-analysis was conducted as comprehensively as possible, several shortcomings and limitations were observed. First, not all patients received two-drug GP regimen. Different chemotherapy regimens result in varying toxicities, which may affect our outcomes for acute adverse reactions. Second, several trials included a short follow-up period. More clinical studies are needed to focus on patients’ long-term survival and the quality of life. Third, all patients in this meta-analysis were Asian, which may be due to high incidence of NPC in Asia. The establishment of prognosis model should be emphasized to screen patients suitable for gemcitabine-based IC regimen chemotherapy. Fourth, considering that this treatment is relatively new in NPC and the toxicity of gemcitabine, only two RCT studies were included in the systematic review. More prospective RCT trials should be conducted to determine the best IC regimen and its dose. Lastly, similar to other meta-analyses, our study featured publication bias.

## 5 Conclusion

Our meta-analysis clearly suggested the feasibility of adding gemcitabine-based IC before CCRT, which might be used as an alternative to IC based on taxane. Hence, we may recommend gemcitabine-based IC plus CCRT as a better choice for LA-NPC patients, but this conclusion should be verified by more high-quality trials.

## Author contributions

HBC and QF conceived and designed the study. SWC, QF, HBC, CMZ, JJX and XH performed the bioinformatics analysis. HBC and QF analyzed the data. HBC and QF wrote the manuscript. SWC and CMZ reviewed and checked the manuscript. All authors read and approved the final manuscript.

**Conceptualization:** Qian Fei.

**Data curation:** qian fei, Jia-Jun Xu.

**Formal analysis:** qian fei, Jia-Jun Xu.

**Funding acquisition:** Song-Wang Chen.

**Investigation:** Chun-Mei Zhang.

**Methodology:** Chun-Mei Zhang.

**Project administration:** Qian Fei.

**Software:** Jia-Jun Xu.

**Writing – original draft:** Qian Fei, Han-Bo Chen.

**Writing – review & editing:** Xia He.
